# An end-to-end LSTM-Attention based framework for quasi-steady-state CEST prediction

**DOI:** 10.3389/fnins.2023.1281809

**Published:** 2024-01-04

**Authors:** Wei Yang, Jisheng Zou, Xuan Zhang, Yaowen Chen, Hanjing Tang, Gang Xiao, Xiaolei Zhang

**Affiliations:** ^1^Great Bay University, Dongguan, China; ^2^College of Engineering, Shantou University, Shantou, China; ^3^School of Mathematics and Statistics, Hanshan Normal University, Chaozhou, China; ^4^Department of Radiology, Second Affiliated Hospital of Shantou University Medical College, Shantou, China

**Keywords:** CEST-MRI, QUASS CEST, deep learning, Bloch-McConnell equation, LSTM-Attention

## Abstract

Chemical exchange saturation transfer (CEST)-magnetic resonance imaging (MRI) often takes prolonged saturation duration (Ts) and relaxation delay (Td) to reach the steady state, and yet the insufficiently long Ts and Td in actual experiments may underestimate the CEST measurement. In this study, we aimed to develop a deep learning-based model for quasi-steady-state (QUASS) prediction from non-steady-state CEST acquired in experiments, therefore overcoming the limitation of the CEST effect which needs prolonged saturation time to reach a steady state. To support network training, a multi-pool Bloch-McConnell equation was designed to derive wide-ranging simulated Z-spectra, so as to solve the problem of time and labor consumption in manual annotation work. Following this, we formulated a hybrid architecture of long short-term memory (LSTM)-Attention to improve the predictive ability. The multilayer perceptron, recurrent neural network, LSTM, gated recurrent unit, BiLSTM, and LSTM-Attention were included in comparative experiments of QUASS CEST prediction, and the best performance was obtained by the proposed LSTM-Attention model. In terms of the linear regression analysis, structural similarity index (SSIM), peak signal-to-noise ratio (PSNR), and mean-square error (MSE), the results of LSTM-Attention demonstrate that the coefficient of determination in the linear regression analysis was at least *R*^2^ = 0.9748 for six different representative frequency offsets, the mean values of prediction accuracies in terms of SSIM, PSNR and MSE were 0.9991, 49.6714, and 1.68 × 10^−4^ for all frequency offsets. It was concluded that the LSTM-Attention model enabled high-quality QUASS CEST prediction.

## 1 Introduction

Chemical exchange saturation transfer (CEST)-magnetic resonance imaging (MRI) of dilute labile protons that undergo their chemical exchange with the bulk water protons enables a specific contrast and provides a promising molecular imaging tool for *in vivo* applications (Zaiss and Bachert, 2013; Xiao et al., 2015; Wu et al., 2016; Jones et al., 2018; Zaiss et al., 2022). However, the CEST effect is limited by experimental conditions such as the amplitude (Sun et al., 2007; Zhao et al., 2011) and duration of RF saturation (Randtke et al., 2014; Zaiss et al., 2018). For some CEST-MRI experiments, the CEST effect needs prolonged saturation duration to achieve quasi-steady-state (QUASS). The limitation of maximum RF saturation duration underestimates the CEST signal (Zhang et al., 2021), which makes it difficult to compare the results between different platforms and stations (Sun, 2021; Wu et al., 2022). So the task for a post-processing strategy to automatically derive the QUASS CEST effect from experimental measurements with limited saturation duration needs to be solved today. Particularly, Sun conducted a QUASS CEST analysis that compensated the effect of finite saturation duration (Ts) and relaxation delay (Td) by solving both the labile proton fraction ratio and exchange rate from simulated CEST, therefore improving the accuracy of CEST-MRI quantification (Sun, 2021). Zhang et al. developed a postprocessing strategy to derive the QUASS CEST by modeling the CEST signal evolution as a function of Ts and Td, allowing robust CEST quantification (Zhang et al., 2021). Kim et al. proposed a QUASS CEST algorithm that can minimize dependences on Ts and Td by combining multi-slice CEST imaging with QUASS processing (Kim et al., 2022).

**Figure 1:**
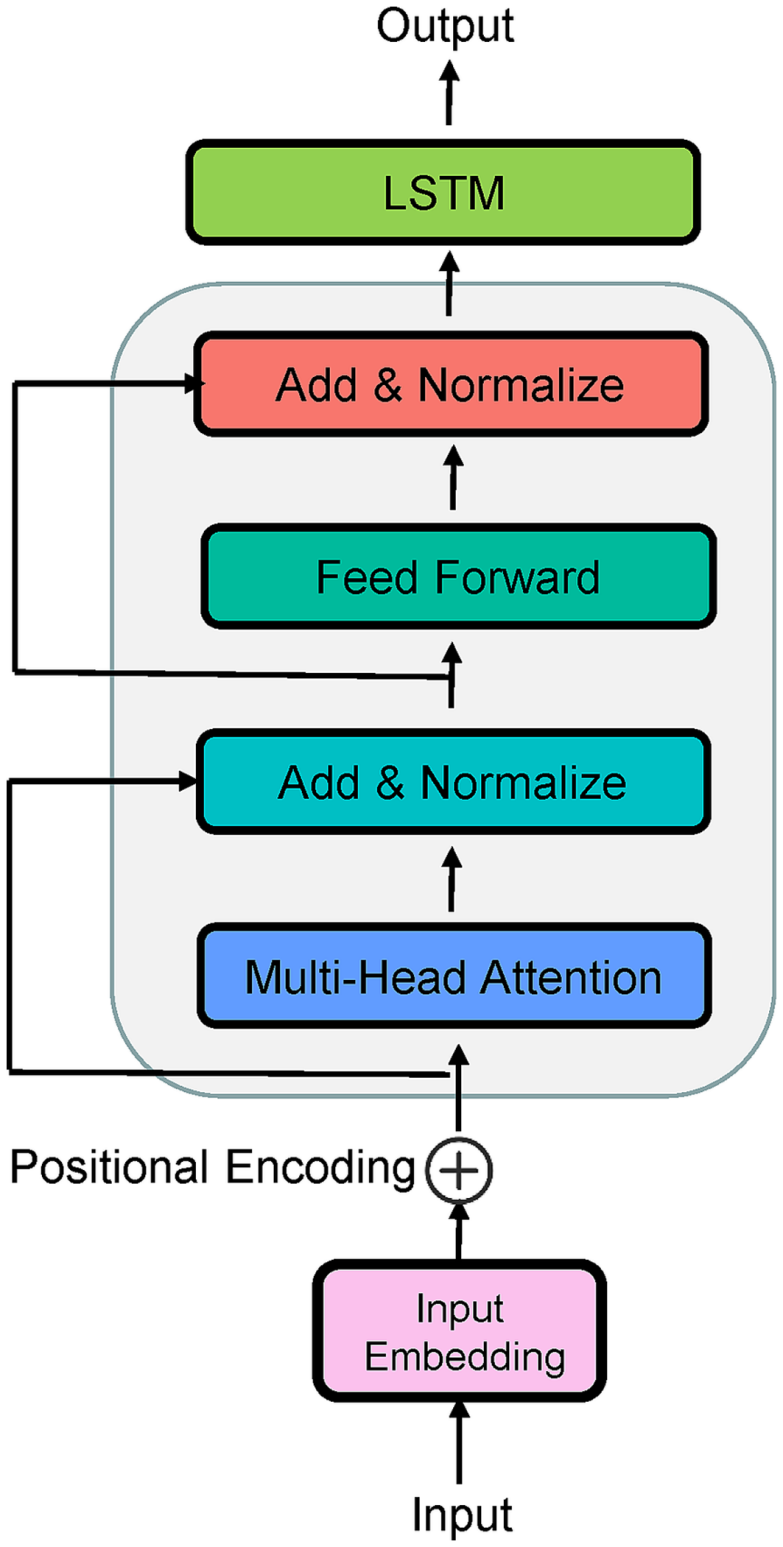
Structure diagram of LSTM-Attention.

The application of deep learning to the CEST-MRI has led to a large number of technical improvements (Glang et al., 2020; Li et al., 2020; Bie et al., 2022; Huang et al., 2022; Perlman et al., 2022), including shortcut of the conventional Lorentzian fitting for *in vivo* 3 T CEST data (Glang et al., 2020), prediction of the CEST contrasts for Alzheimer’s disease (Huang et al., 2022), identification of pertinent Z-spectral features for distinguishing tumor aggressiveness (Bie et al., 2022), etc. Therefore, this paper aims to employ a deep learning technique to predict QUASS CEST (i.e., CEST images on prolonged saturation) by training a network on the prior knowledge of simulated CEST Z-spectra. With respect to the underlying application domain, the sequence-to-sequence (Seq2Seq) network is an intuitive approach, in which the LSTM (Yu et al., 2019) and the attention mechanism (Vaswani et al., 2017) are stated as excellent methods. LSTM is known to solve the vanishing gradient problem when a recurrent neural network (RNN) is used to work with the sequence input, while the disadvantage of LSTM in the latent decomposition training is significant (Shi et al., 2022). For this, some modified versions help improve the LSTM performance and were successfully applied to medical treatment behavior prediction (Cheng et al., 2021), medical event prediction (Liu et al., 2022), and EEG-based emotion recognition (Chakravarthi et al., 2022). Particularly, a Seq2Seq with a multi-head attention mechanism instead of recurrence has excelled at tasks of time series, obtaining effective information and significant spatiotemporal features from the new coding sequence. However, the attention mechanism loses the sequential information because the used attention mechanism is position-insensitive (Zheng et al., 2021). The principles of the LSTM network and attention mechanism were briefly described in Supplementary File 1. Looking at the advantages and disadvantages of both algorithms, a hybrid model named LSTM-Attention would be a perfectly natural way. In this LSTM-Attention architecture, the LSTM is used to obtain the hidden state of the input features, while the use of multi-head attention in the encoder layer is to better learn the temporal information (Figure 1). In Figure 1, the input embedding is used to capture high-dimensional spatial properties of long time series. Because position information is not considered in the attention layer, we add “positional encoding” to the input embeddings. To this end, different semantic information from different sequence positions is incorporated into an embedding tensor, compensating for the lack of position information. The LSTM had a hidden state dimensionality of 1,024, the number of attention heads is 4.

**Figure 2:**
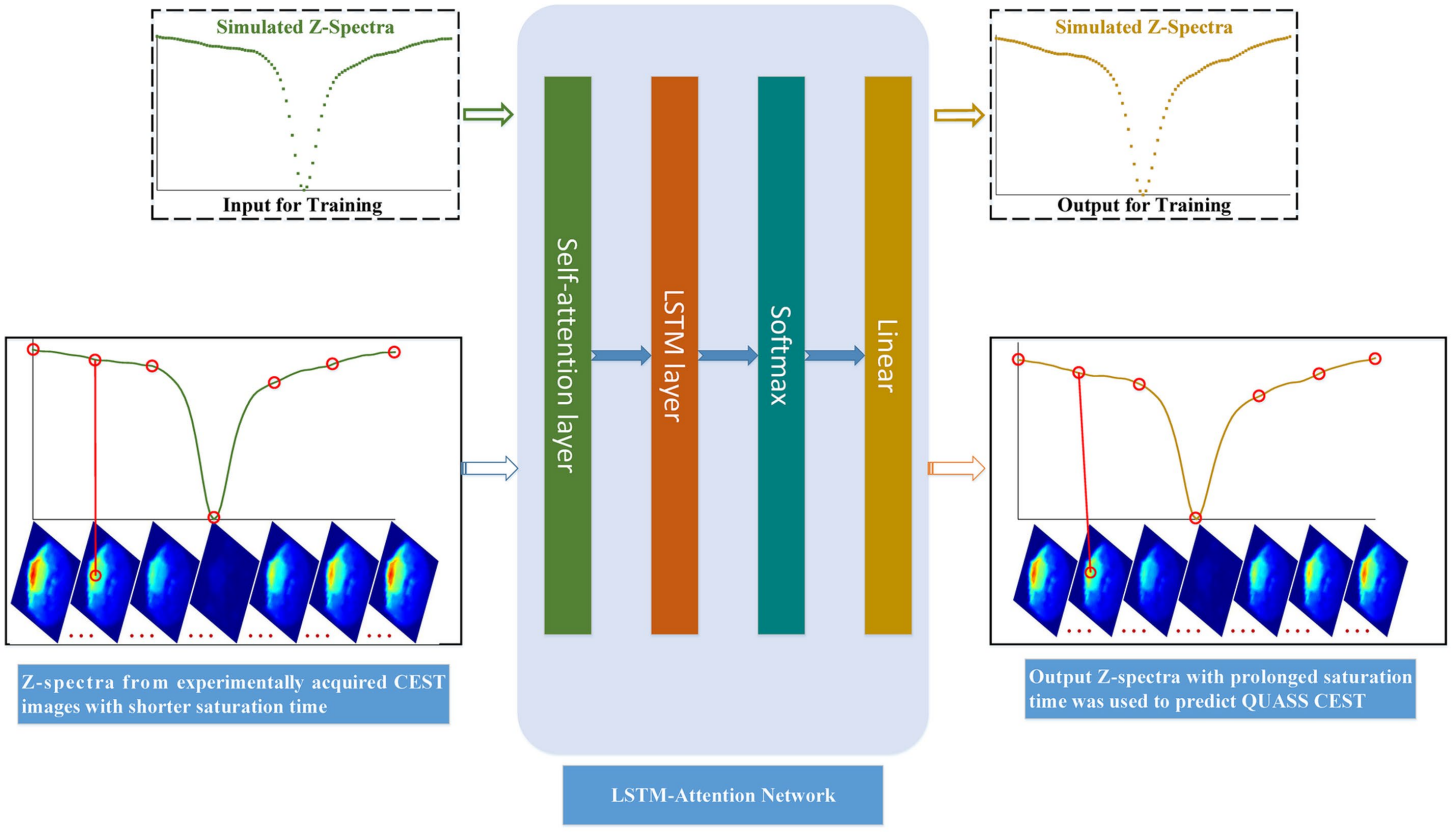
Flow chart of LSTM-Attention based model for predicting QUASS CEST. The simulated Z-spectra with short saturation time was referred to the training input, and the simulated Z-spectra with prolonged saturation time was the training output. Input Z-spectra from experiments with shorter saturation times for each image pixel, we can predict QUASS CEST images by their output Z-spectra of each pixel.

Motivated by the above, this paper aims to build an LSTM-Attention-based model for QUASS CEST prediction from non-steady-state CEST (i.e., CEST images with shorter saturation time) acquired in experiments, as shown in Figure 2. Simulated Z-spectra with shorter and prolonged saturation time was derived from the designed Bloch–McConnell equations (Xiao et al., 2023), respectively. Then we used the trained model to predict QUASS CEST from non-steady-state CEST acquired in experiments.

In summary, this work makes the following two key contributions. To tackle the problematic and time-consuming task of obtaining the labeled training data from experiments, we built a large-scale training set based on simulated Z-spectra derived from the designed Bloch-McConnell equations. We formulated an LSTM-Attention-based model which is trained on simulated CEST Z-spectra to predict QUASS CEST image pixel-by-pixel from non-steady-state CEST acquired in explements, where the attention mechanism improves the predictive ability of LSTM by paying attention to the input weights that contribute more to the output.

## 2 Materials and methods

### 2.1 *In vivo* MRI experiments

In this vivo MRI experiment, 8-week-old male SD rats (Beijing Vital River Laboratory Animal Technology Co., Ltd.) weighing 250 g were used to generate a tumor-bearing model. All animal care and experimental procedures were performed in accordance with the National Research Council Guide for the Care and Use of Laboratory Animals. For this assessment, a 10 μL suspension of rat glioma C6 cells (approximately 2 × 106 cells) was implanted into the right basal ganglia (specific injection position: AP + 1, ML + 3, DV-5) of the rats using a Hamilton syringe and a 30-gauge needle. Two weeks after the implantation of tumor cells, the rats were subjected to MRI.

The CEST-MRI experiment was performed using a 7 T horizontal bore small animal MRI scanner (Agilent Technologies, Santa Clara, CA, U.S.A.) with a surface coil (Timemedical Technologies, China) for transmission and reception. Imaging parameters were as follows: repetition time (TR) = 6,000 ms, echo time (TE) = 40 ms, array = frequency offsets, slice thickness = 2 mm, field of view (FOV) = 64 × 64 mm, matrix size = 64 × 64, spatial resolution = 1 × 1 mm, averages = 1. To obtain CEST images, an echo planar imaging readout sequence was used, where continuous wave (CW) RF irradiation was implemented on scanners. The saturation times were 1.5 s and 5 s, respectively, with 101 frequency offsets evenly distributed from −6 to 6 ppm relative to the resonance of water.

The CEST images of saturation times 1.5 s acquired in this experiment were the inputs of trained networks. The CEST images with saturation times 5 s acquired in this experiment were the reference, which is used to assess the prediction performance by comparing the model’s estimates with the experimental data values.

### 2.2 Training dataset

The training of LSTM-Attention for predicting objects requires a large dataset with true pixel-level labels in terms of saturation times, which is extremely expensive to construct training data in experiments. To address this issue, we simulated CEST signals using a 7-pool Bloch–McConnell equation (Xiao et al., 2023) at both non-steady and quasi-steady states. This 7-pool model consists of free water centered at 0 ppm, amide centered at 3.5 ppm, guanidyl/amine centered at 2.0 ppm, hydroxyl centered at 1.3 ppm, nuclear Overhauser enhancement (NOE) centered at-1.6 ppm, magnetization transfer (MT) centered at-2.4 ppm, and NOE centered at-3.5 ppm. In detail, 20 dynamic parameters regarding all possible tissue combinations were considered. For each dynamic parameter, random variables from the uniform distribution with lower bound and upper bound were sampled for the training dataset, so we could generate as much data as needed with all possible tissue combinations. The sampled variables of each parameter interacting with that of each other generated 350,000 parameter combinations, thus yielding 350,000 paired simulated Z-spectra (see Supplementary Figure S1). The simulated Z-spectra with saturation times of 1.5 s and 5 s at 101 offsets in the range of ±6 ppm were referred to as the training input and output, respectively.

### 2.3 Evaluation metrics and workstation

Linear regression analysis (Li et al., 2020) was first applied to evaluate the proposed model at frequency offsets-3.48 ppm, −2.40 ppm, −1.56 ppm, 1.32 ppm, 2.04 ppm, and 3.48 ppm. To evaluate the proposed model in the prediction of CEST image at each frequency offset, the prediction performance was evaluated by three measures: the structural similarity index (SSIM), the peak signal-to-noise ratio (PSNR) (Hore and Ziou, 2010), and mean squared error (MSE).

The workstation used in this study is a Lenovo ST558 workstation with 32 G memory, a dual-core CPU10 core, and a 2.4 G main operating frequency. The experiments are based on PyTorch, and the number of epochs is 100. The number of batch size is 256. The optimizer is Adam, and the learning rate is 0.0001. We initialize the weights using samples from a uniform distribution, and use MSE-Loss as the loss function.

## 3 Results

To validate the proposed model, prediction images were compared with the reference from experimental measurements. We applied the trained neural networks to predict the CEST images with a saturation time of 5 s from experimentally acquired CEST images with a saturation time of 1.5 s. For comparison, the LSTM-Attention presented comparable performance to that of five popular existing networks: the multilayer perceptron (MLP) (Xu et al., 2018), recurrent neural network (RNN) (Xu et al., 2018), long short-term memory (LSTM) (Yu et al., 2019), gated recurrent unit (GRU) (Xu et al., 2018), and BiLSTM (Siami-Namini et al., 2019).

**Figure 3:**
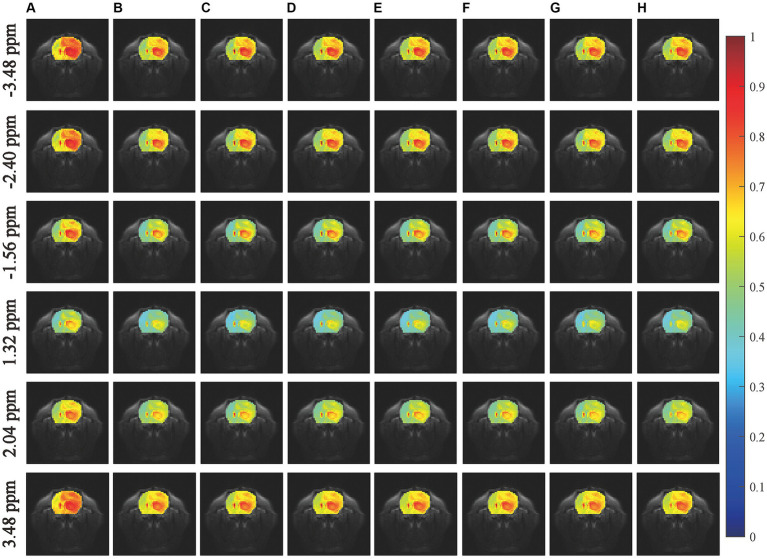
Comparisons of the predicted results with experimentally acquired CEST images at frequency offsets-3.48 ppm, −2.40 ppm, −1.56 ppm, 0.96 ppm, 2.04 ppm, and 3.48 ppm. The column **(A)** shows the experimentally acquired CEST image with the saturation time of 1.5 s, the column **(B)** shows the experimentally acquired CEST image with the saturation time of 5 s (reference), the columns **(C–H)** denote the prediction results obtained by MLP, RNN, LSTM, GRU, BiLSTM and LSTM-Attention, respectively.

We first conducted an experiment to predict CEST images at frequency offsets-3.48 ppm, −2.40 ppm, −1.56 ppm, 1.32 ppm, 2.04 ppm, and 3.48 ppm, as shown in Figure 3. The region of the pseudo color image overlaid on the anatomy image was the region of interest (ROI). The results obtained from the considered networks were almost equivalent to those obtained experimentally by the subjective vision.

**Figure 4:**
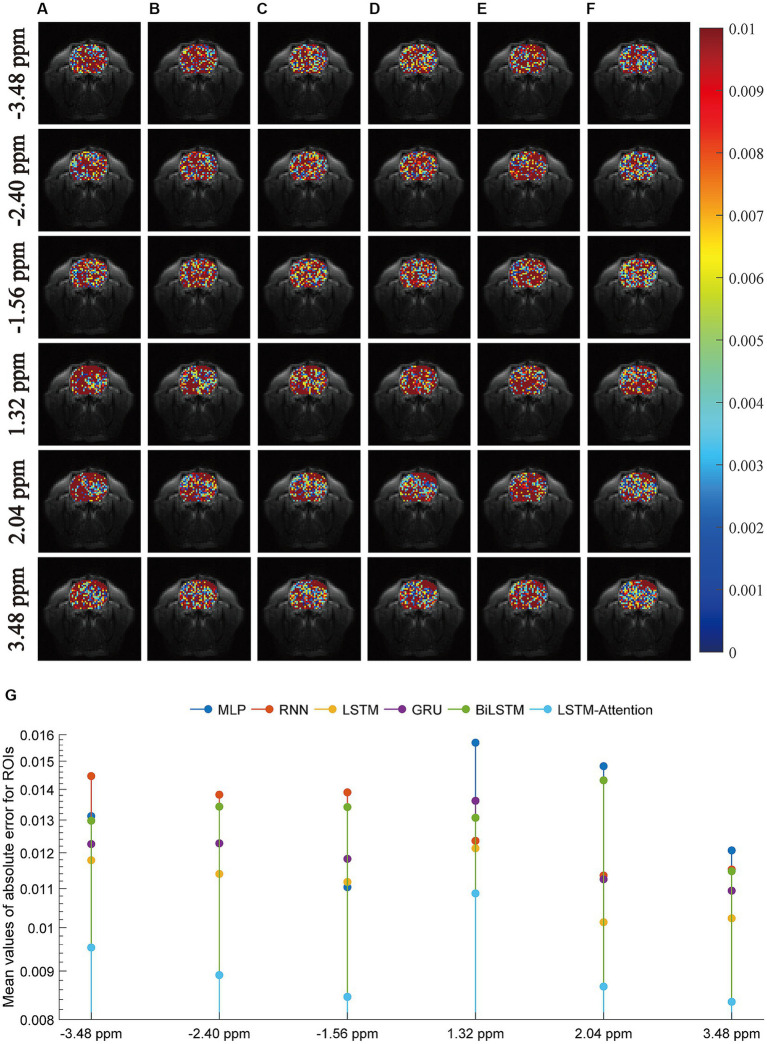
The absolute error modulus between the predicted images and the experimentally acquired CEST images at frequency offsets-3.48 ppm, −2.28 ppm, −1.56 ppm, 1.32 ppm, 2.04 ppm, and 3.48 ppm. The columns **(A–F)** are the results from MLP, RNN, LSTM, GRU, BiLSTM and LSTM-Attention, respectively; the plot **(G)** denotes the mean values of absolute error modulus obtained by considered six networks.

Furthermore, we carried out a comparison experiment in terms of the absolute error modulus between reference and prediction, as illustrated in Figure 4. In this figure, row-plots indicated the absolute error modulus at frequency offsets −3.48 ppm, −2.40 ppm, −1.56 ppm, 1.32 ppm, 2.04 ppm, and 3.48 ppm; columns (A–F) were the absolute error modulus from MLP, RNN, LSTM, GRU, BiLSTM and LSTM-Attention, respectively; the plot (G) denoted the mean values of absolute error modulus at frequency offsets −3.48 ppm, −2.40 ppm, −1.56 ppm, 1.32 ppm, 2.04 ppm and 3.48 ppm that obtained by considered methods. The results of Figure 4G reveal that the mean values of absolute error modulus obtained from the proposed LSTM-Attention model are smaller than those of other networks at these frequency offsets, while there is a difference of one order of magnitude between the LSTM-Attention and its counterparts at frequency offsets-3.48 ppm, −2.40 ppm, −1.56 ppm, 2.04 ppm, and 3.48 ppm. In other words, the CEST image at these frequency offsets obtained by the trained LSTM-Attention showed a higher degree of agreement with those obtained by the experimental measurements as the standard.

**Figure 5:**
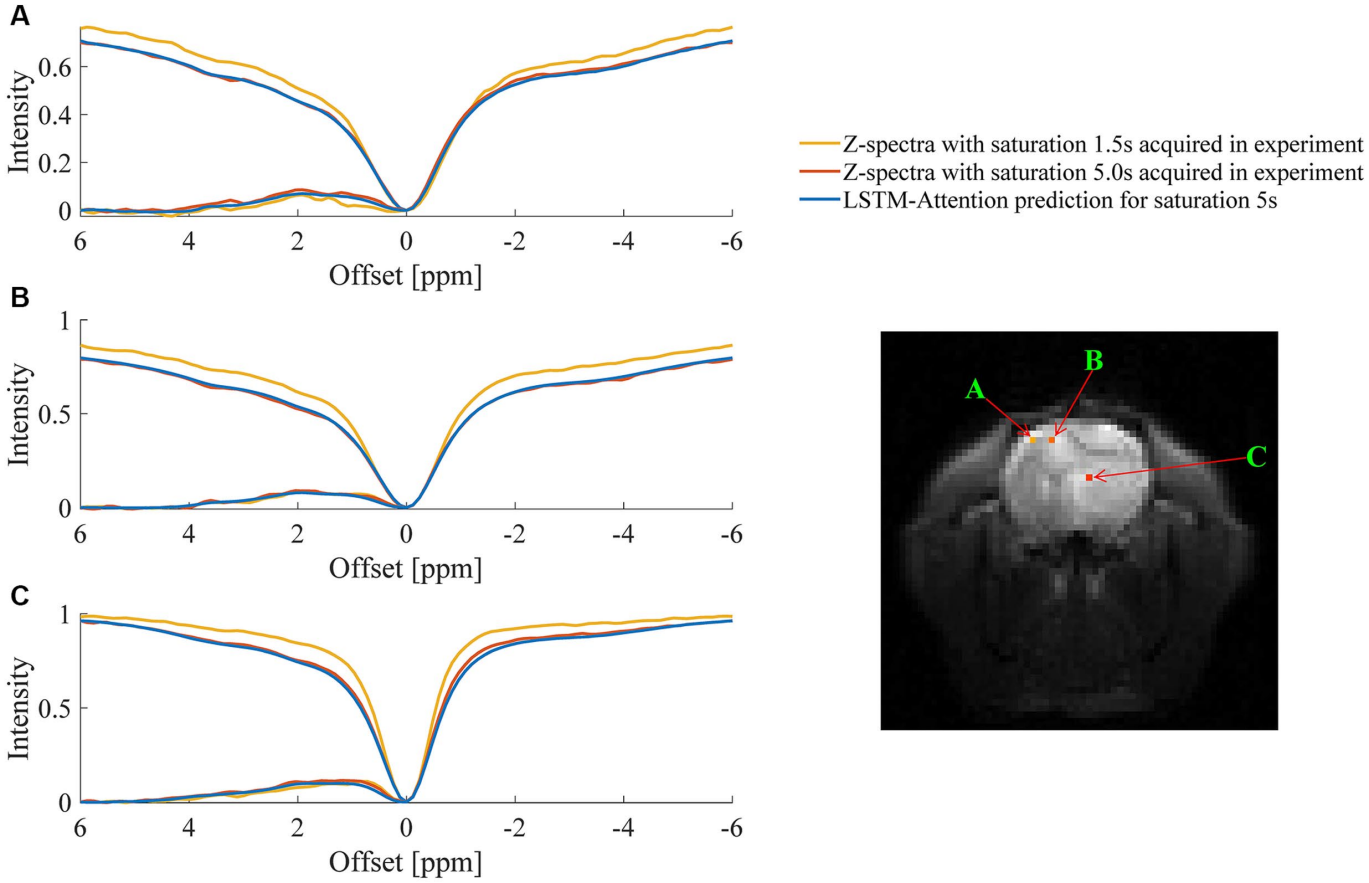
Comparison between the predicted Z-spectra of LSTM-Attention and the experimentally acquired results at one randomly chosen pixel of **(A)** gray matter, **(B)** white matter and **(C)** tumor, respectively.

An example of the predicted Z-spectra by LSTM-Attention for white matter, gray matter, and tumor is shown in Figure 5, which consistently provided satisfactory results.

**Figure 6:**
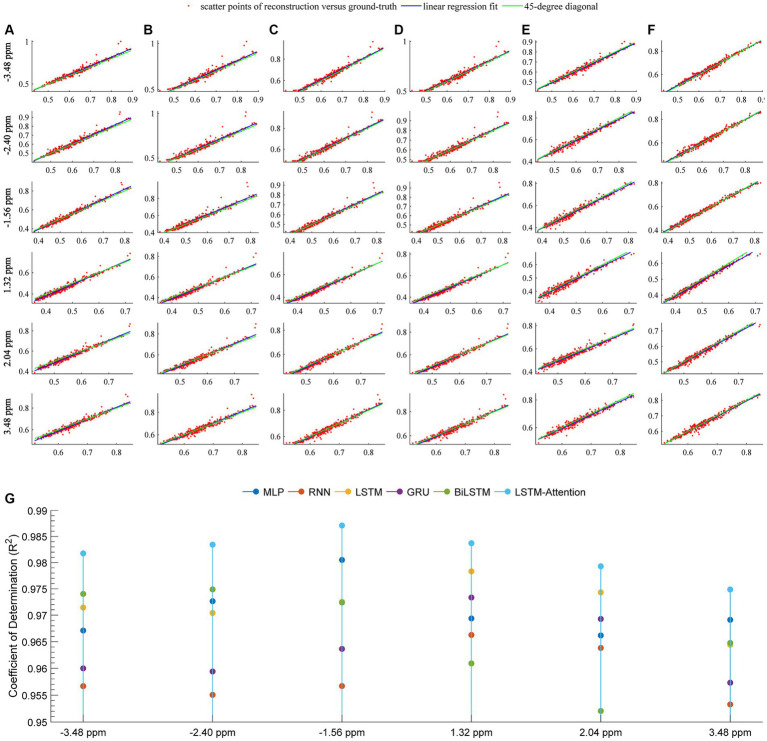
Linear regression analysis of the prediction and reference at frequency offsets-3.48, −2.40, −1.56, 1.32, 2.04, 3.48 ppm. The columns **(A–F)** are the results from MLP, RNN, LSTM, GRU, BiLSTM and LSTM-Attention, respectively; the plot **(G)** denotes the coefficient of determination (*R*^2^) between the prediction and reference. For the columns **(A–F)** at each offset, the locations of the red markers are specified by the vectors *x* and *y*, where *x* is the pixel values of the experimentally acquired CEST image with the saturation time 5 s (reference) and y is the pixel values of predicted CEST images with a saturation time 5 s; the blue line is the linear regression fitting based on the red scatter points of prediction versus reference, the green line indicates the 45-degree diagonal.

Figure 6 quantitatively demonstrates the considered networks for predicting the *in vivo* CEST signal by plotting the linear regression lines and scatter diagrams between the reference and the prediction. In this figure, row-plots were the results at frequency offsets −3.48 ppm, −2.40 ppm, −1.56 ppm, 1.32 ppm, 2.04 ppm, and 3.48 ppm; columns (A–F) denoted the results from MLP, RNN, LSTM, GRU, BiLSTM and LSTM-Attention, respectively; the plot (G) denoted the coefficient of determination at frequency offsets −3.48 ppm, −2.40 ppm, −1.56 ppm, 1.32 ppm, 2.04 ppm, and 3.48 ppm that obtained by considered methods. The pixel values correspond to the points of the ROI in Figures 3, 4. For each plot, the fitting curve was denoted by the blue line and the green line was the 45-degree diagonal. The excellent performance of our prediction was confirmed by the scatter and linear regression lines, resulting in a very high coefficient of determination (*R*^2^ ≥ 0.9748) at these frequency offsets.

**Figure 7:**
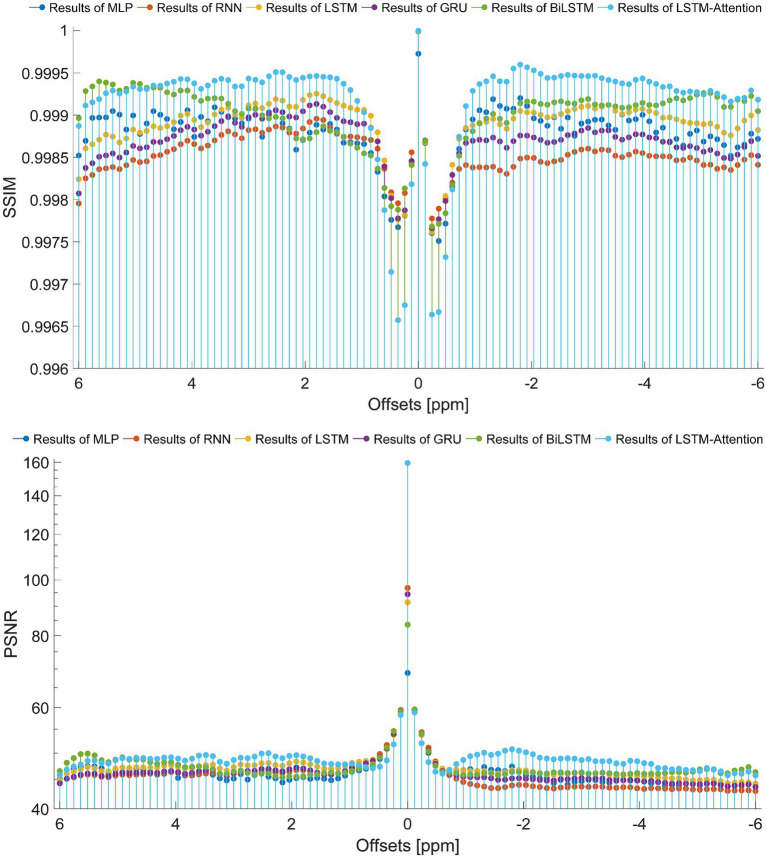
The SSIM and PSNR obtained from the reference and the prediction at each offset (−6 ~ 6 ppm).

**Figure 8:**
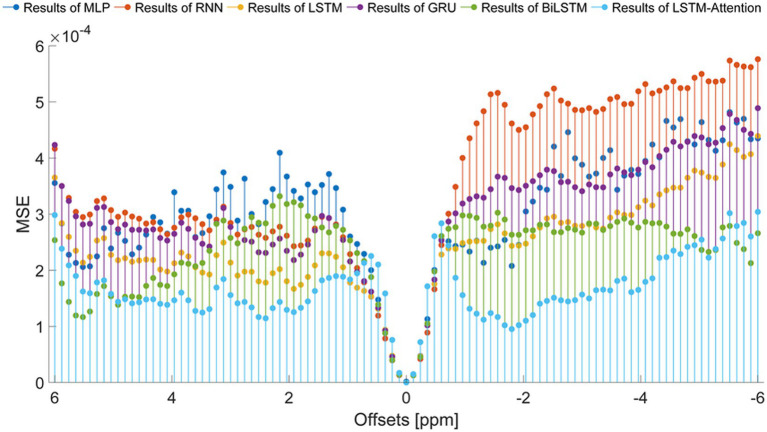
The MSE obtained from the reference and the prediction at each offset (−6 ~ 6 ppm).

To set up a comprehensive way to evaluate the performance of the prediction models, the SSIM and PSNR from the reference and the prediction at each offset (−6 ~ 6 ppm) are considered, as displayed in Figure 7. In terms of SSIM, the LSTM-Attention exhibits good accuracies at each offset (−6 ~ 6 ppm) and presents results close to those of LSTM at-5.04 and 0.96 ppm, while it exceeds the performance of other networks in the ranges (−6 ~ −5.16 ppm), (−4.92 ~ −0.72 ppm) and (1.08 ~ 4.68 ppm). Similar results are obtained by LSTM-Attention in terms of PSNR. Clearly, our model exhibits competitive results for these two metrics based on different criteria, providing a mean SSIM value of 0.9991 and a mean PSNR value of 49.6714, respectively. Figure 8 displays the MSE obtained by considered networks for all frequency offsets, and the best result of mean MSE 1.68 × 10^−4^ is obtained by the LSTM-Attention network.

## 4 Discussions

To some extent, we developed a general deep learning-based approach to predict QUASS CEST using experimentally acquired CEST images with shorter saturation times, since the performances of MLP and five existing Seq2Seq networks are also evaluated in this study. As the results show, the LSTM-Attention network outperforms the MLP, RNN, LSTM, GRU, and BiLSTM (Figure 7). It is clear that LSTM-Attention is able to capture the underlying context better by paying attention to the input weights that contribute more to the output. The better performance of LSTM-Attention compared to its counterparts is understandable for certain types of data such as specific chemical groups in the downfield and MT/NOE in the upfield (Figures 4, 6).

In fact, the Z-spectra of a pixel typically behaves short-and long-range dependencies along the frequency offsets (see Supplementary Figure S2). The LSTM-Attention is consistently the best model followed by MLP, RNN, LSTM, GRU, and BiLSTM for capturing the short-and long-range behavior. In the simplest form, fully RNN is an MLP with the previous set of hidden unit activations feeding back into the network along with the inputs (Roy et al., 2019). Additionally, the LSTM, GRU, BiLSTM, and LSTM-Attention are able to overcome RNN’s vanishing gradient problem which happens when RNN learns long-range dependencies of inputs (Yang et al., 2022). Therefore, the ability of short-and long-range interaction in these considered networks performs similarly, as the results above. Particularly, the LSTM-Attention augments the non-linear processing capability in QUASS CEST prediction by taking advantage of the known, observed, and static covariate factors.

In practice, training a deep neural network to predict QUASS CEST requires massive samples with ground-truth annotations, which is extremely expensive to construct experimentally. To solve this problem, we built an automatically labeled dataset based on the Bloch–McConnell equations. Briefly, we considered all the possible parameters of the equations when generating the trained samples. For each dynamic parameter, a wide range of random values was sampled in a uniform distribution with its lower and upper bounds, automatically yielding a large set of labeled training data.

Further studies would be beneficial for QUASS CEST applications at low-field MRI where short saturation time is needed. It could be useful to investigate other less visible CEST effects (such as guanidyl or amine) in clinical MRI scanners.

## 5 Conclusion

In summary, we addressed the QUASS CEST predicting problem in learning systems and proposed a data-driven predicting scheme that benefits from our strategy to reduce the effect of finite RF saturation duration on the CEST measurement. The experiment study compared the proposed model with other approaches, and the effectiveness and superiority of the LSTM-Attention model were validated. This research can be further expanded to predict problems for available clinical MRI scanners.

## Data availability statement

The raw data supporting the conclusions of this article will be made available by the authors, without undue reservation.

## Ethics statement

The animal study was approved by National Research Council Guide for the Care and Use of Laboratory Animals. The study was conducted in accordance with the local legislation and institutional requirements.

## Author contributions

WY: Formal analysis, Methodology, Software, Visualization, Writing – original draft. JZ: Data curation, Formal analysis, Software, Visualization, Writing – original draft. XuZ: Data curation, Formal analysis, Software, Visualization, Writing – original draft. YC: Funding acquisition, Investigation, Project administration, Resources, Writing – review & editing. HT: Data curation, Resources, Visualization, Writing –review & editing. GX: Formal analysis, Investigation, Methodology, Software, Supervision, Validation, Writing – review & editing. XiZ: Conceptualization, Funding acquisition, Investigation, Methodology, Project administration, Supervision, Validation, Writing – review & editing.
