# Supplementary figures and images for: An end-to-end LSTM-Attention based framework for quasi-steady-state CEST prediction
Source: Front Neurosci. 2024 Jan 4;17:1281809. doi: 10.3389/fnins.2023.1281809 (PMC10797904; doi:10.3389/fnins.2023.1281809)

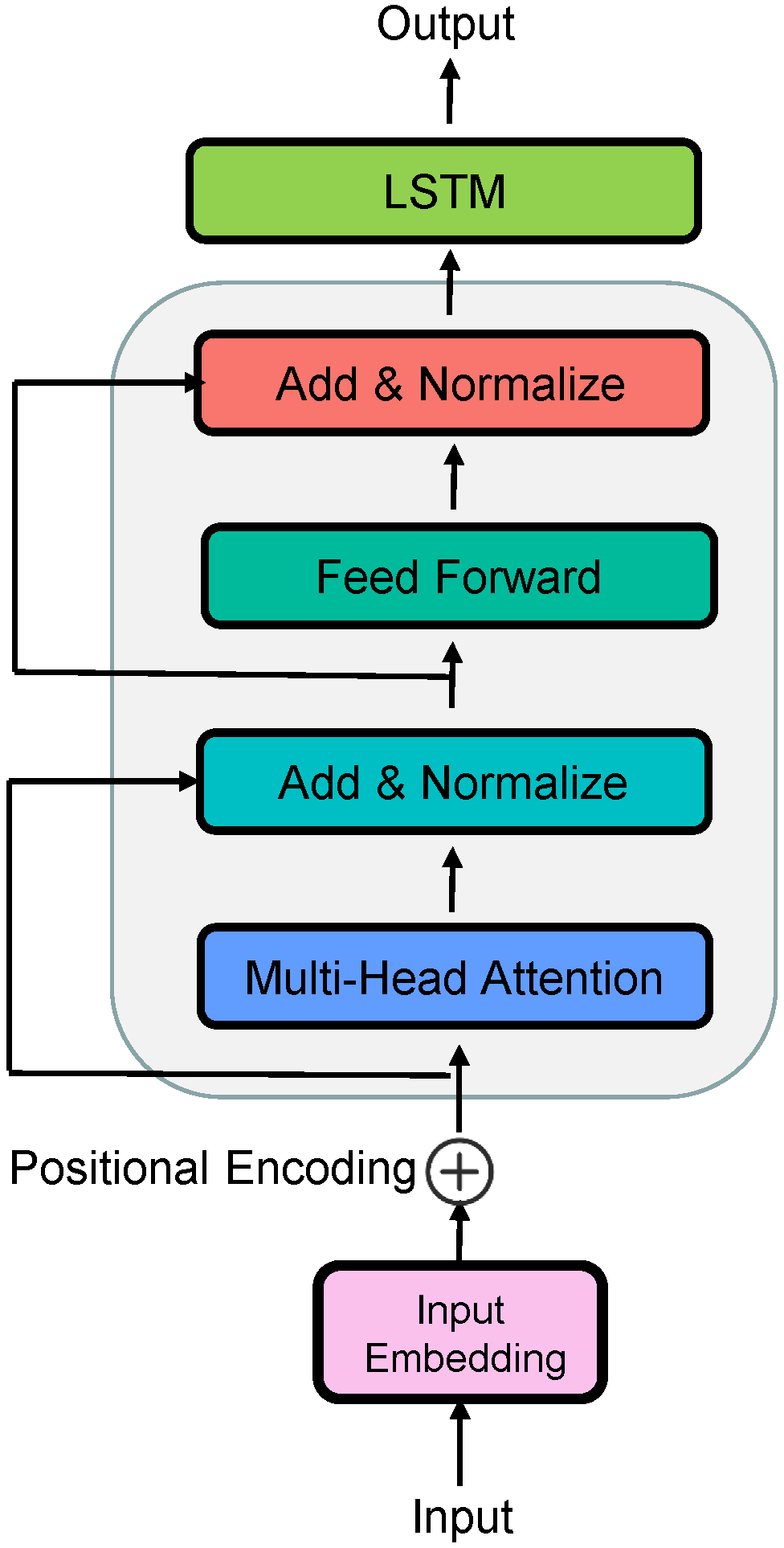

Supplement: Supplementary file 1 [file Figure_1.TIF]

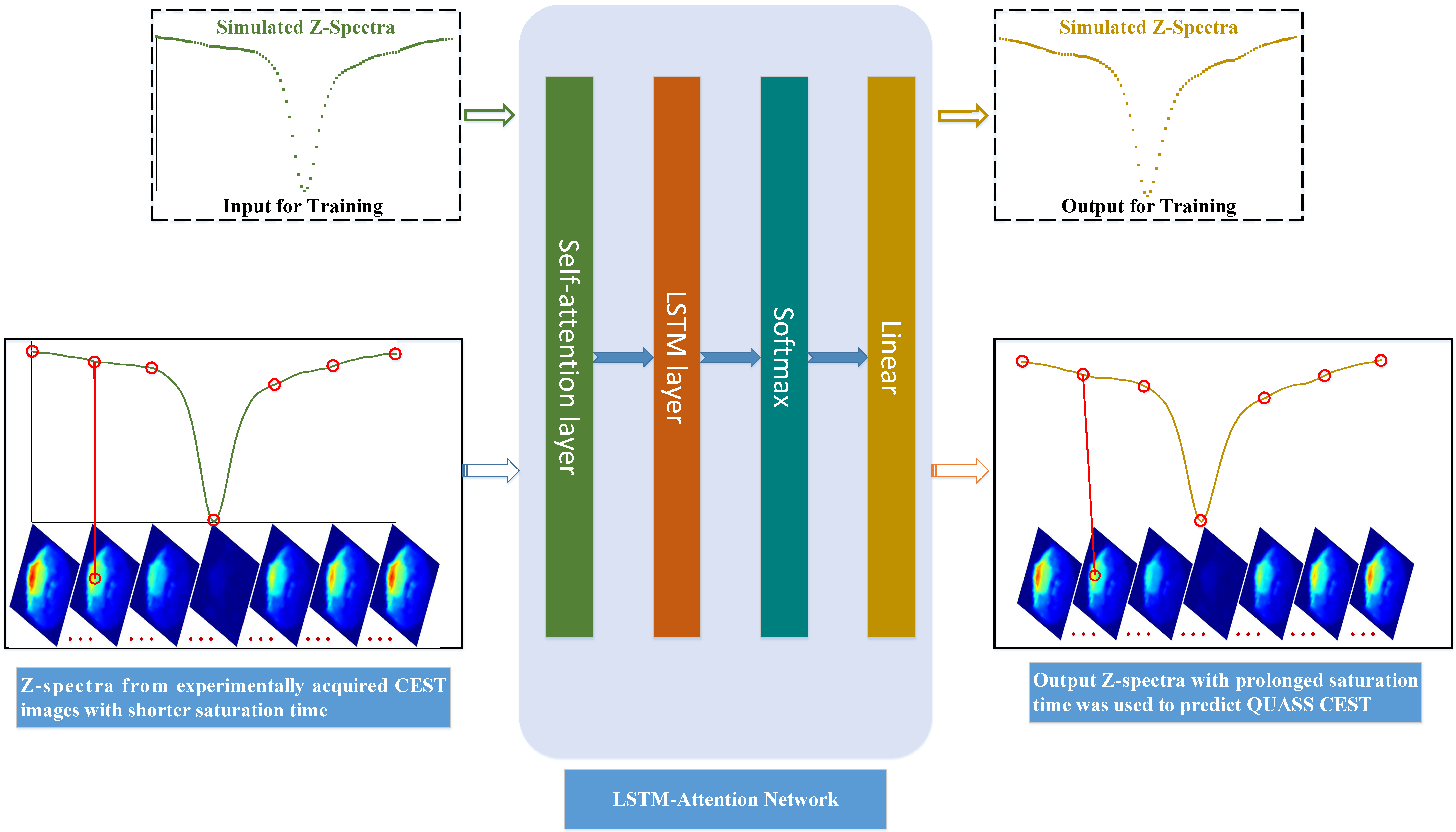

Supplement: Supplementary file 2 [file Figure_2.TIFF]

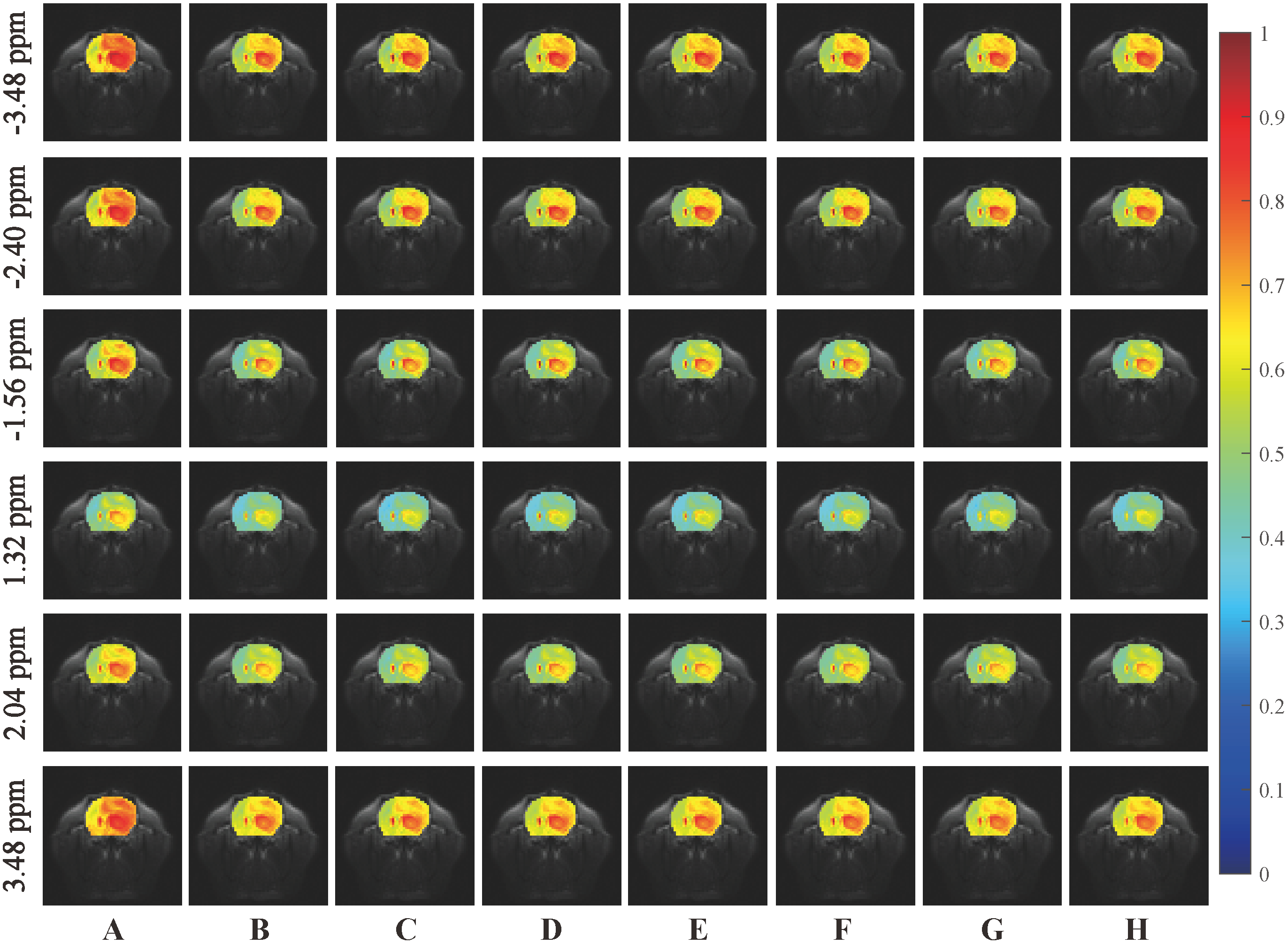

Supplement: Supplementary file 3 [file Figure_3.TIFF]

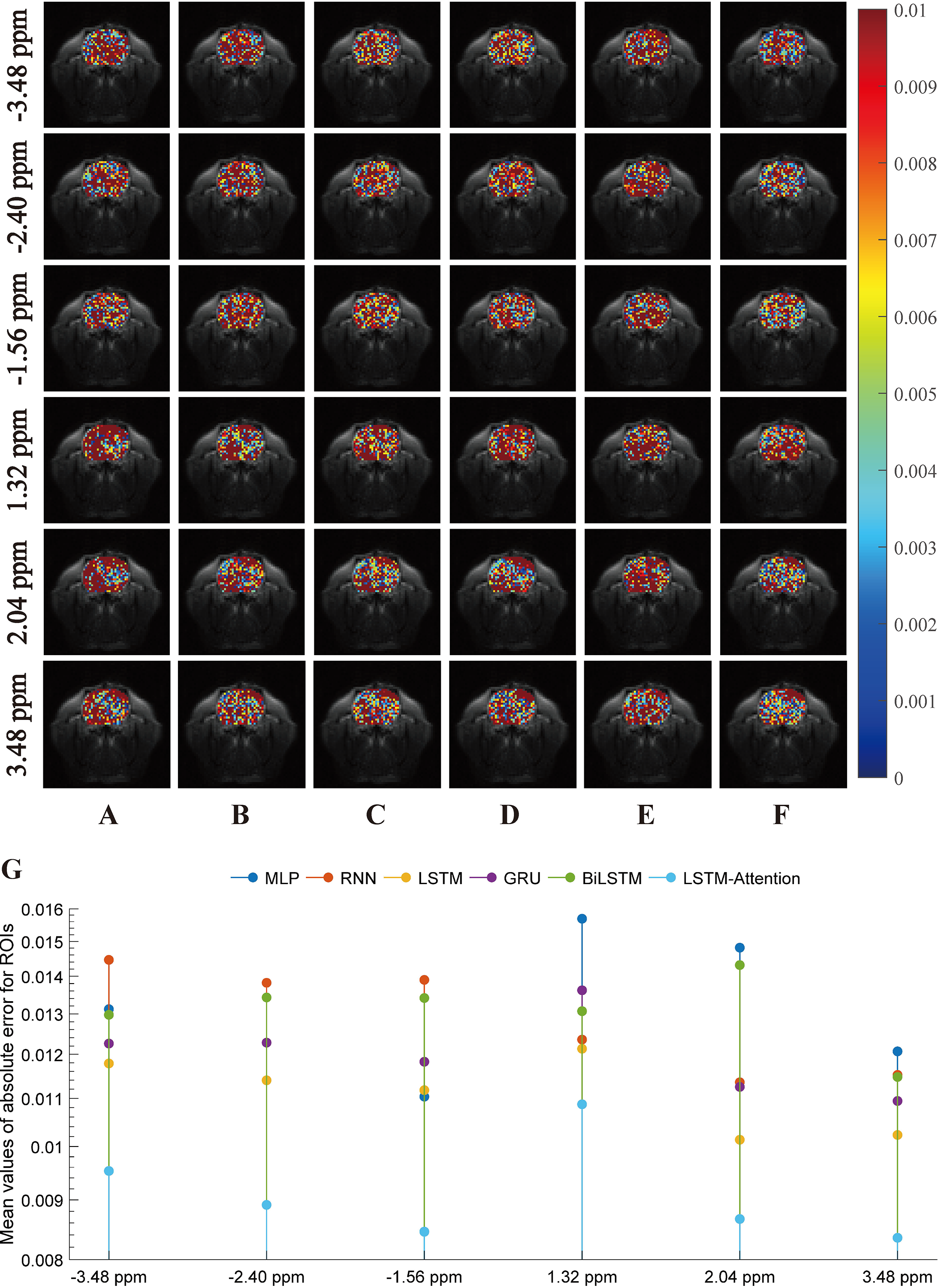

Supplement: Supplementary file 4 [file Figure_4.TIF]

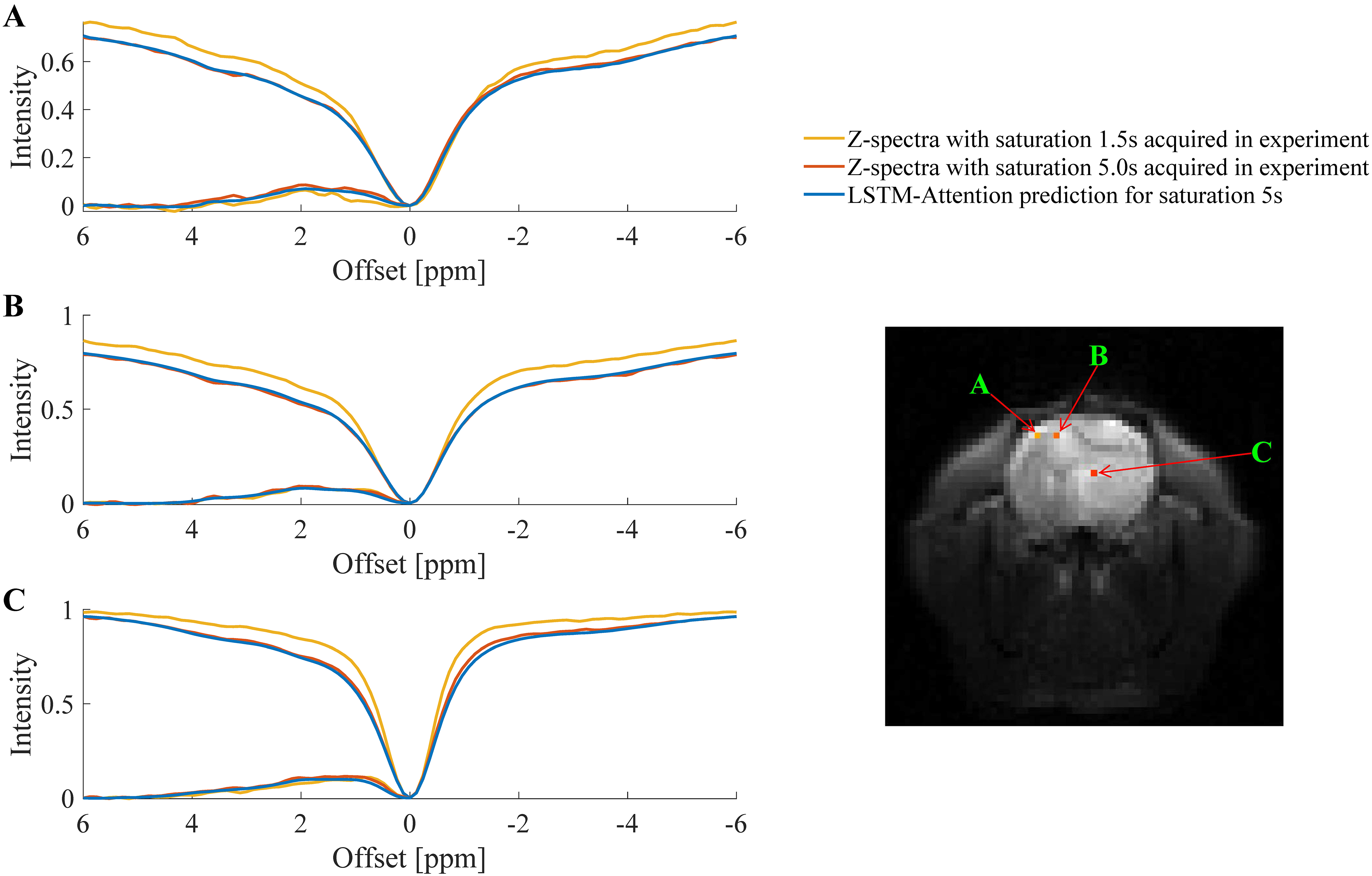

Supplement: Supplementary file 5 [file Figure_5.TIF]

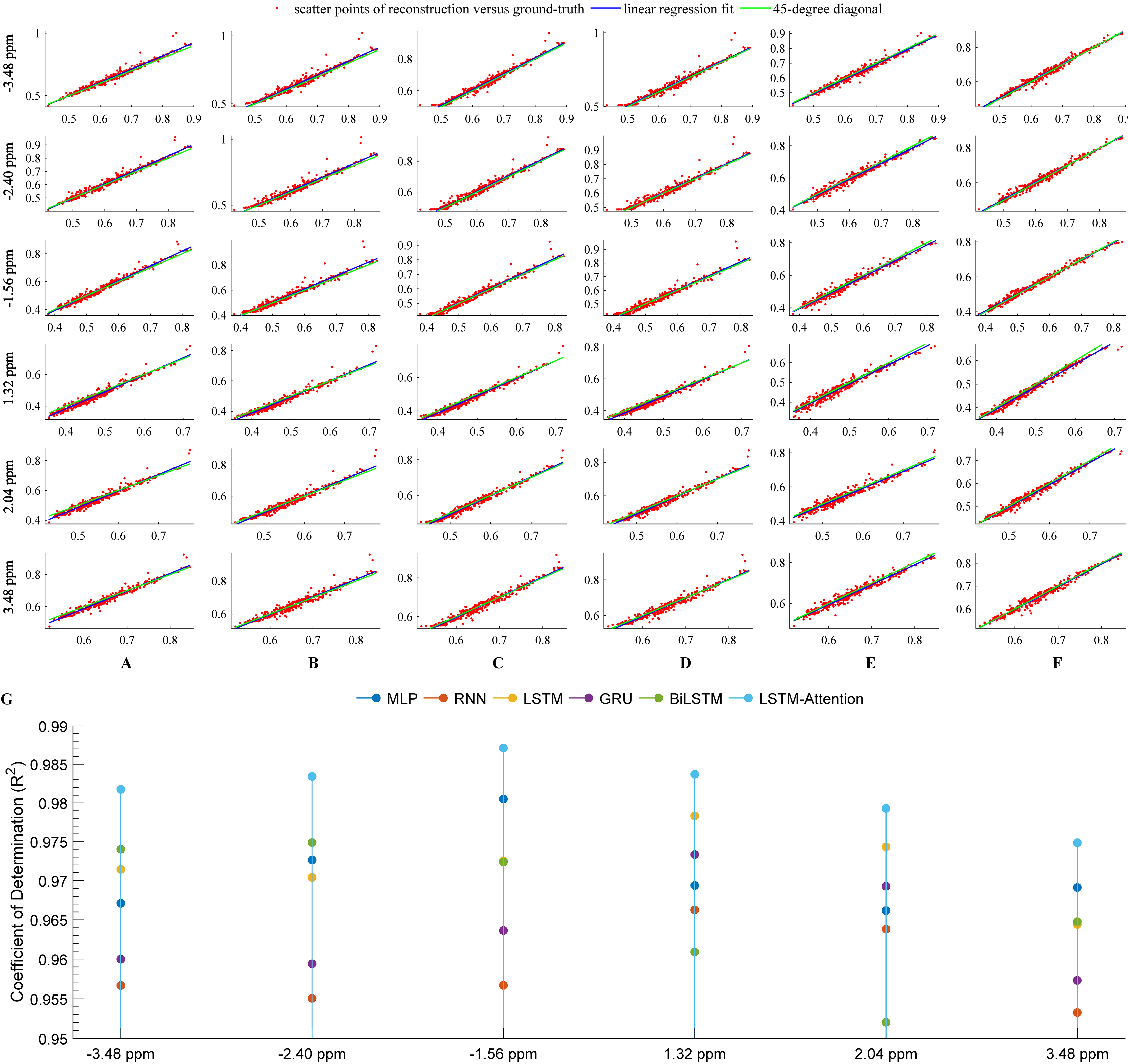

Supplement: Supplementary file 6 [file Figure_6.TIF]

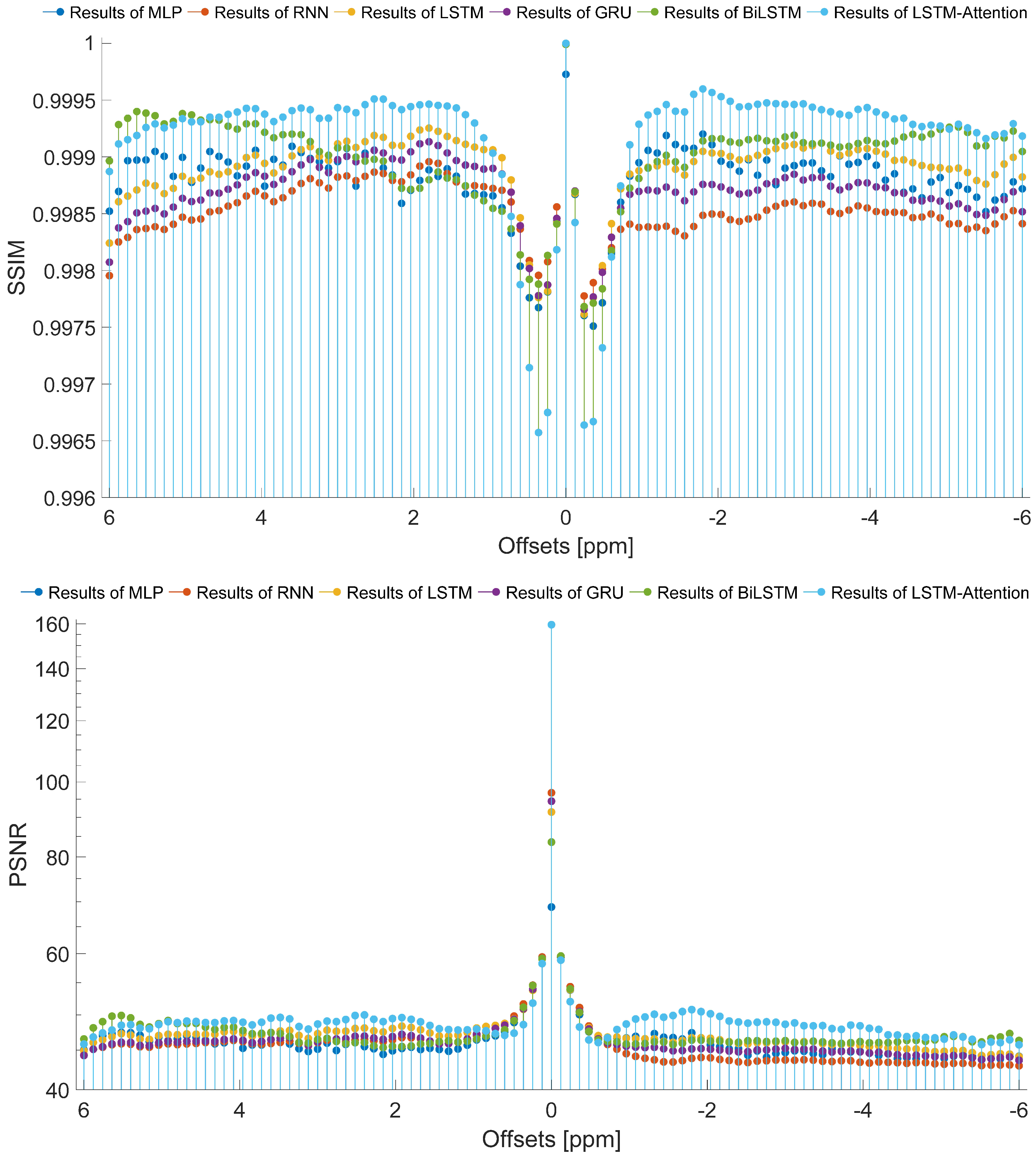

Supplement: Supplementary file 7 [file Figure_7.TIF]

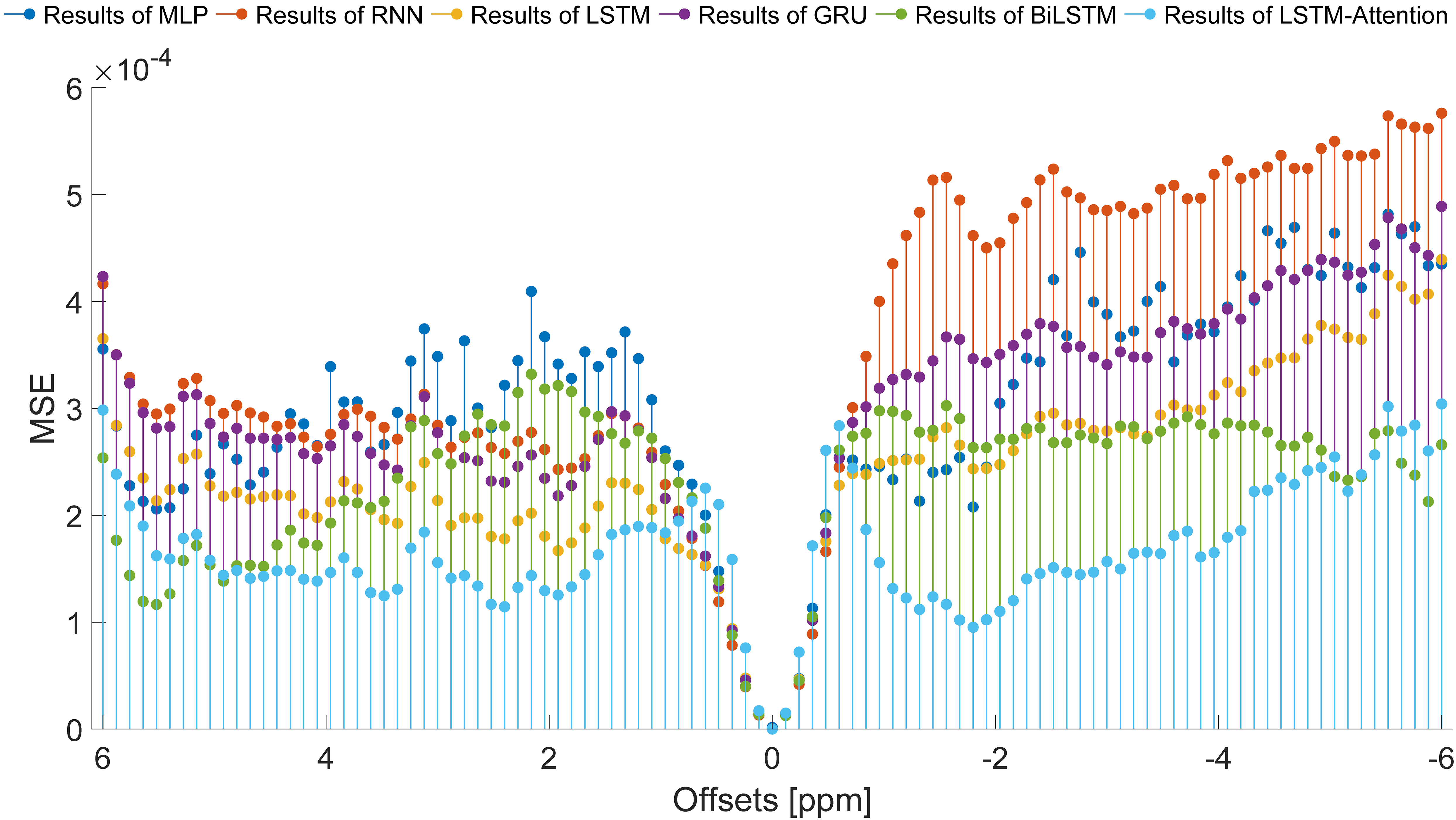

Supplement: Supplementary file 8 [file Figure_8.TIFF]
