# Supplementary material for: An end-to-end LSTM-Attention based framework for quasi-steady-state CEST prediction
Source: Front Neurosci. 2024 Jan 4;17:1281809. doi: 10.3389/fnins.2023.1281809 (PMC10797904; doi:10.3389/fnins.2023.1281809)

Supplementary Material

1 Supplementary File 1

A. LSTM

Essentially, LSTM is a recurrent neural network for processing time series data. LSTM model solves the gradient disappearance problem of RNNs by learning the long-term dependencies between time series. In practice, LSTM replaces the hidden layer of RNNs with a memory unit, which is controlled by a forget gate, an input gate and an output gate (see Plot 1). By using this memory unit, it can automatically select the best lag time and selectively remember historical information, thus solving the gradient disappearance problem caused by the long sequence of RNN.

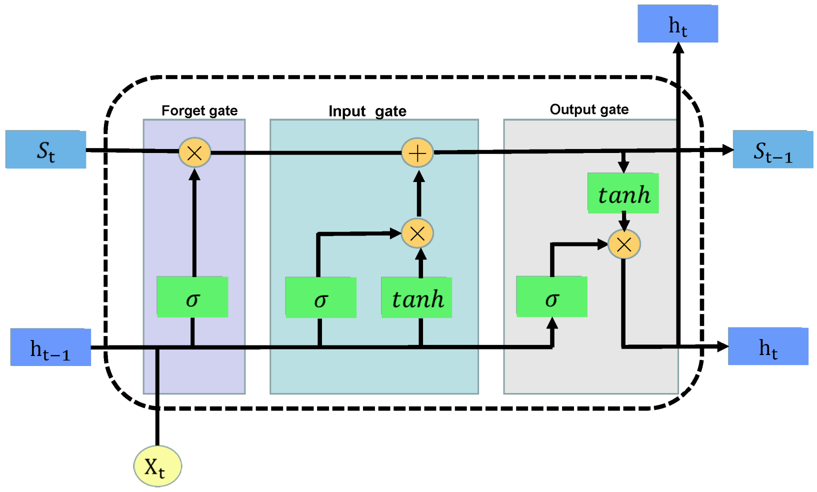

Plot 1: LSTM memory unit.

B. Attention Mechanism

The attention mechanism follows this overall using stacked self-attention and point-wise, fully connected layers for both the encoder and decoder.

The core functionality of the Attention network is the multi-head attention. An attention function can be described as mapping a query and a set of key-value pairs to an output, where the query, keys, values, and output are all vectors. The attention head evaluates an output score. We compute the score as:

$$Attention(Q, K, V) = softmax(\frac{QK^T}{\sqrt{d_k}})$$

Multi-head attention allows the model to jointly attend to information from different representation subspaces at different positions. With a single attention head, averaging inhibits this.

$$MultiHead(Q,K,V) = Concat(head_1, head_2, \cdots head_h)W$$

$$where\ head_i = Attention(QW, KW, VW)$$

where the projections are parameter matrices W.

2 Supplementary Figures

Supplementary Figure 1. The visualization of simulated Z-spectra with saturation 1.5 s and 5.0 s at 101 offsets in the range of ± 6 ppm.

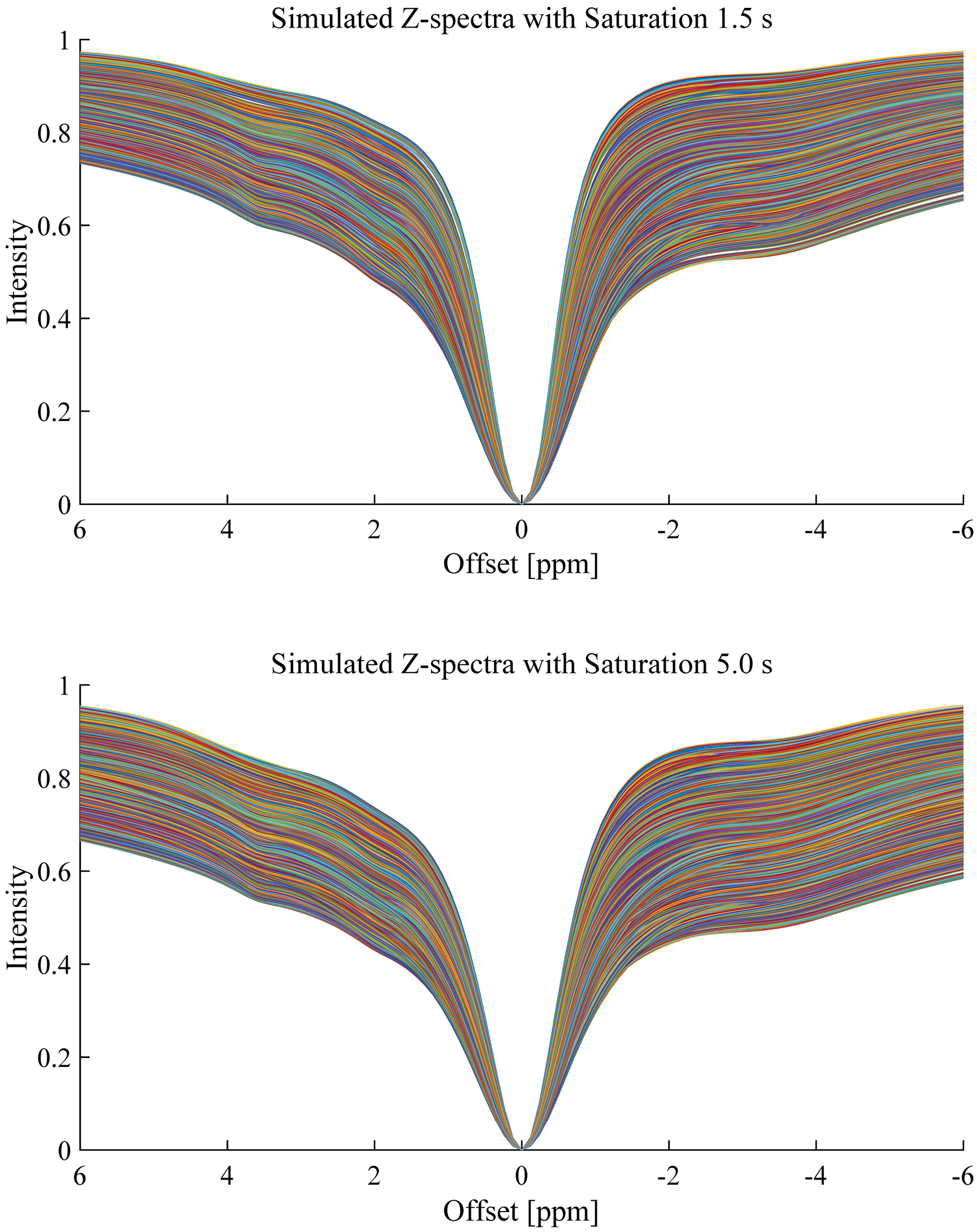

Supplementary Figure 2. The heatmap of correlation coefficients matrix for experimentally acquired CEST images at frequency offsets [-6, 6] ppm.

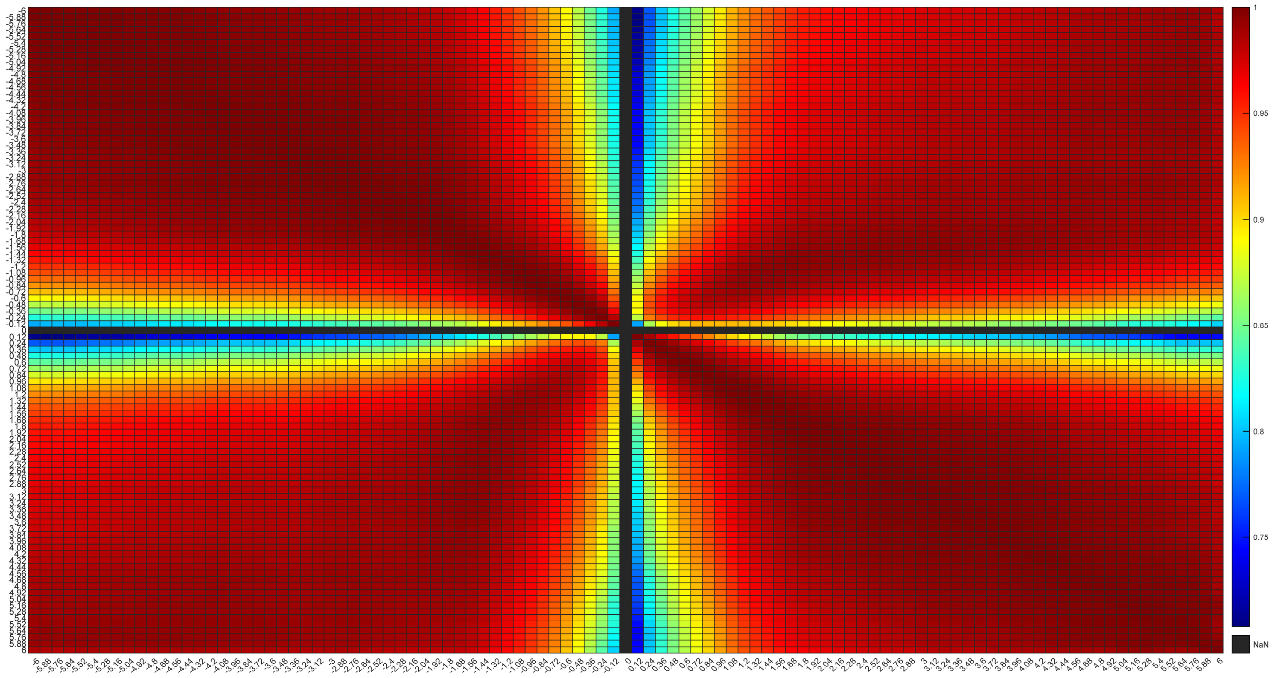

Supplement: Supplementary file 9 [file Data_Sheet_1.PDF]
